# IDENTIFYING RESILIENT PERSONALITY THROUGH LATENT PROFILE ANALYSIS IN SEXUAL MINORITY MIDDLE-AGED AND OLDER ADULTS

**DOI:** 10.1093/geroni/igad104.2571

**Published:** 2023-12-21

**Authors:** Christi Nelson

**Affiliations:** University of Washington, Seattle, Washington, United States

## Abstract

Research has found evidence of resilience in sexual minority (i.e., lesbian, gay, bisexual) adults, despite experiencing higher rates of poorer health and facing additional stressors such as discrimination and victimization. Yet, very limited research has been conducted on resilience in this marginalized group. Utilizing data from the Midlife in the United States study, this study conducted latent profile analysis (LPA) using the Big Five personality traits (i.e., openness, conscientiousness, extraversion, agreeableness, neuroticism) to identify distinct personality profiles in a sample of sexual minority and propensity-score matched heterosexual middle-aged and older adults (n=477, mean age= 57.3, SD= 10.9 years, age range= 40-83 years). Additionally, the associations between the personality profiles and health-risk/promoting behaviors were assessed. Four profiles were identified: Average (35.2%), Resilient (47%), Overcontrolled (7.3%), and Undercontrolled (10.5%). The results found that Resilient sexual minority participants were significantly more likely to engage in problematic drinking but were also more likely to have a routine physical exam than Resilient heterosexual participants. Both sexual minority and heterosexual participants with a Resilient personality profile were more likely to engage in moderate physical activity than their counterparts with other personality profiles. These results suggest that sexual minority adults have the potential for resilience which is often overlooked in research. This is one of the first studies to use LPA to identify personality profiles in a sample with a focus on sexual minority participants. Most previous research using LPA to identify personality profiles did not include data on sexual orientation.

